# Syncope Diagnosis at Referral to a Tertiary Syncope Unit: An in-Depth Analysis of the FAST II

**DOI:** 10.3390/jcm12072562

**Published:** 2023-03-29

**Authors:** Jelle S. Y. de Jong, Steven van Zanten, Roland D. Thijs, Ineke A. van Rossum, Mark P. M. Harms, Joris R. de Groot, Richard Sutton, Frederik J. de Lange

**Affiliations:** 1 Department of Clinical and Experimental Cardiology, Heart Center, Amsterdam Cardiovascular Sciences, Amsterdam UMC, University of Amsterdam, 1105 AZ Amsterdam, The Netherlands; 2 Department of Cardiology, Reinier de Graaf Gasthuis, 2625 AD Delft, The Netherlands; 3 Stichting Epilepsie Instellingen Nederland (SEIN), 2103 SW Heemstede, The Netherlands; 4 Department of Neurology, Leiden University Medical Centre, 2333 ZA Leiden, The Netherlands; 5 Department of Internal and Emergency Medicine, University Medical Centre Groningen, University of Groningen, 9713 GZ Groningen, The Netherlands; 6 Department of Cardiology, National Heart & Lung Institute, Hammersmith Hospital Campus, Imperial College, London SW7 6LY, UK

**Keywords:** syncope, guideline implementation, diagnostic accuracy, diagnostic yield, transient loss of consciousness

## Abstract

Objective: A substantial number of patients with a transient loss of consciousness (T-LOC) are referred to a tertiary syncope unit without a diagnosis. This study investigates the final diagnoses reached in patients who, on referral, were undiagnosed or inaccurately diagnosed in secondary care. Methods: This study is an in-depth analysis of the recently published Fainting Assessment Study II, a prospective cohort study in a tertiary syncope unit. The diagnosis at the tertiary syncope unit was established after history taking (phase 1), following autonomic function tests (phase 2), and confirming after critical follow-up of 1.5–2 years, with the adjudicated diagnosis (phase 3) by a multidisciplinary committee. Diagnoses suggested by the referring physician were considered the phase 0 diagnosis. We determined the accuracy of the phase 0 diagnosis by comparing this with the phase 3 diagnosis. Results: 51% (134/264) of patients had no diagnosis upon referral (phase 0), the remaining 49% (130/264) carried a diagnosis, but 80% (104/130) considered their condition unexplained. Of the patients undiagnosed at referral, three major causes of T-LOC were revealed: reflex syncope (69%), initial orthostatic hypotension (20%) and psychogenic pseudosyncope (13%) (sum > 100% due to cases with multiple causes). Referral diagnoses were either inaccurate or incomplete in 65% of the patients and were mainly altered at tertiary care assessment to reflex syncope, initial orthostatic hypotension or psychogenic pseudosyncope. A diagnosis of cardiac syncope at referral proved wrong in 17/18 patients. Conclusions: Syncope patients diagnosed or undiagnosed in primary and secondary care and referred to a syncope unit mostly suffer from reflex syncope, initial orthostatic hypotension or psychogenic pseudosyncope. These causes of T-LOC do not necessarily require ancillary tests, but can be diagnosed by careful history-taking. Besides access to a network of specialized syncope units, simple interventions, such as guideline-based structured evaluation, proper risk-stratification and critical follow-up may reduce diagnostic delay and improve diagnostic accuracy for syncope.

## 1. What Is Already Known on This Topic

Many syncope patients remain undiagnosed, resulting in inappropriate therapy or no therapy at all, and thereby a lower quality of life, and are referred to the tertiary syncope unit, despite workup with many diagnostic tests in primary or secondary care.

Since outcomes in the follow-up to assess and confirm diagnosis is considered the gold standard for syncope, the use of long-term follow-up, including an expert committee, is the most feasible way to confirm accuracy of syncope diagnosis.

### 1.1. What This Study Adds

For the first time, an in-depth analysis is performed of which patient diagnoses remain undiagnosed or inaccurately diagnosed by referring physicians, and how these diagnoses could be established in a tertiary referral syncope unit.

### 1.2. How This Study Might Affect, Research, Practice or Policy 

This study shows the difficulties and errors among referrals to a tertiary syncope unit, and indicates how these problems may be overcome.

This study fills the knowledge gap in diagnosing simple and complex syncope patients.

## 2. Introduction

Transient loss of consciousness (T-LOC) is a condition characterized by a sudden and temporary loss of consciousness and postural tone, with spontaneous and complete recovery. Patients with T-LOC constitute a common clinical problem and account for 1–3% of emergency department visits. Of these, up to 40% result in hospitalization [1]. T-LOC is associated with a decreased quality of life and high healthcare costs [2]. Once their presenting symptoms are correctly diagnosed, patients can receive adequate treatments and counselling regarding the cause of syncope, which may reduce the burden of T-LOC on them [3]. Estimates of the diagnostic yield of the initial evaluation vary, but approximately 40% of patients with T-LOC remain undiagnosed in the emergency department and in secondary care [4]. It could be hypothesized that after consultation with multiple specialists in secondary care, establishing a diagnosis in the tertiary syncope unit becomes increasingly difficult. However, the contrary is the case, as we recently found that in a tertiary syncope unit most patients can still be diagnosed by thorough history-taking [5]. This highlights the importance of a comprehensive structural clinical evaluation in the diagnosis of T-LOC. The complexity of T-LOC lies in the heterogeneity of the underlying etiologies, as the causes of T-LOC lie in the domain of internists, neurologists and cardiologists [6]. Some causes of T-LOC are assigned to a specific specialization (e.g., cardiac arrhythmias to cardiology, epileptic seizures to neurology), while causes arising from pathophysiological changes in short-term blood pressure regulation (non-cardiac syncope diagnoses) are not specifically allocated to any single specialty. Specialized syncope units were created to fill in this gap in medical care [6]. They are outpatient clinics with staff dedicated to patients with T-LOC. Additionally, syncope units have the availability of performing specific tests that mainly emphasizes the analysis of the short-term blood pressure regulation, e.g., autonomic function tests [4]. The use of autonomic function tests is often limited to specialized syncope units. Unavailable in emergency departments or secondary care, these tests, although available in tertiary care, only marginally contributed to the diagnostic yield [5]. The fact that history-taking proved to be the main diagnostic test, even in tertiary care, for establishing a diagnosis of T-LOC [5], raised the following question: which patients referred to the tertiary syncope unit were undiagnosed or inaccurately diagnosed, and subsequently sub-optimally treated in primary/secondary care? Next, we investigated the confirmed diagnosis in relation to the referral diagnosis, and uncovered both underdiagnosis and overdiagnosis of different causes of T-LOC.

## 3. Methods

This is an analysis of the previously published prospective cohort study “Fainting Assessment Study II (FAST II)” [5]. The study was conducted according to the guidelines of the Declaration of Helsinki and approved by the Medical Ethical Committee of the Amsterdam University Medical Centre, location Meibergdreef 9, Amsterdam. A waiver was issued for obtaining informed consent, as the data were collected as part of routine clinical care (ref. W12_172, date: 30 July 2012).

### 3.1. Patients

We included all consecutive patients, 18 years and older, and with at least one T-LOC episode presenting to the syncope unit of the Amsterdam University Medical Centre, at Meibergdreef 9, Amsterdam, between October 2012 and February 2015. 

The FAST II consisted of three active phases, as published [5]. For this analysis “phase 0” diagnosis was added as the referral diagnosis. 

#### 3.1.1. Phase 0 Diagnosis: Suggested Referral Diagnosis

The phase 0 diagnosis was derived from the referral letter to the syncope unit. We considered any suggested diagnosis by the referring physician’s diagnosis. That diagnosis was labelled as the phase 0 diagnosis. In the absence of a specified diagnosis in the referral letter the patient was considered undiagnosed.

#### 3.1.2. Workflow and Diagnostic Phases in the Specialized Syncope Unit

Patients were evaluated at the tertiary syncope unit according to the European Society of Cardiology (ESC) guidelines on syncope [6], and as previously described (de Jong et al. [5]). The consultation consisted of an initial evaluation (phase 1), followed by autonomic function tests, when appropriate (phase 2). The diagnosis was made according to the diagnostic criteria as described in the ESC guidelines, and diagnostic certainty was established according to Table 1 (adopted from Fainting Assessment Study II [5]). Patients could have multiple diagnoses if different causes were identified for different T-LOC episodes. The number of diagnoses, therefore, exceeded the number of patients. During phase 0 and phase 1, we recorded all the diagnostic tests, therapeutic interventions, admissions and consultations that patients underwent following the index T-LOC episode, prior to the consultation.

After one-and-a-half to two years of follow-up, patients received a questionnaire, wherein the patients were asked about T-LOC recurrences, diagnostic tests, admissions, and changes in diagnoses between phase 1 and phase 2. The final phase 3 diagnosis was established by a multidisciplinary expert committee (critical follow-up), which considered the correct diagnoses (gold standard).

Follow-up time was defined as the time from first consultation, to the return of the last questionnaire in days.

### 3.2. Outcomes

Our primary outcomes were (1) the diagnostic accuracy of those with a diagnosis upon referral (i.e., the concordance of phase 0 and phase 3 diagnoses), and (2) the final (phase 3) diagnosis of those who remained undiagnosed in secondary care. The secondary outcome was time-to-diagnosis. Time-to-diagnosis was defined as the days between the index T-LOC episode and time of diagnosis. In patients without a phase 0 diagnosis or with an incorrect phase 0 diagnosis, the syncope unit diagnosis was used as the day of diagnosis. We could not estimate the time to diagnosis in the case of correct phase 0 diagnosis. 

### 3.3. Statistical Analysis

The means with standard deviations were used in the case of normally distributed data, and medians with interquartile range for variables with a non-normal distribution. We calculated ninety-five percent confidence intervals using Wilson’s method to express diagnostic accuracy. Non-normally distributed, (semi) continuous data were compared using a Mann–Whitney U test. Proportions were compared with a Chi-square test. In the case of frequencies below ten in contingency tables, we used a Fisher’s exact test. A *p*-value of less than 0.05 was considered significant. Data were visualised in a transition plot (also called a Sankey diagram).

### 3.4. Patient and Public Involvement

Patients were actively involved during the consultation for feedback and follow-up regarding the evaluation of the syncope consultation at the tertiary referral clinic. 

## 4. Results

### 4.1. Patient Characteristics

A total of 264 patients were included in this analysis. The baseline characteristics upon referral are listed in Table 2.

### 4.2. Phase 0 Diagnoses

The referring physician did not suggest a phase 0 diagnosis in 134 of 264 (51%) patients, while a diagnosis was suggested in the remaining 130 patients (49%) (Table 2). The phase 0 referring physician diagnosis was inaccurate or incomplete in 65% (84 out of 130 patients). Interestingly, 80% of the 130 patients with a phase 0 diagnosis considered their condition to be unexplained. Table 3 shows a detailed overview of patients who were unaware of their suggested diagnosis from the referral physician (phase 0). Of the 134 patients without a phase 0 diagnosis, 132 (99%) stated that their goal of the consultation with the syncope unit was reaching a diagnosis. One patient reported a “second opinion” and one patient an “additional explanation”.

### 4.3. Phase 3 Diagnosis in Patients without a Phase 0 Diagnosis

**Figure 1:**
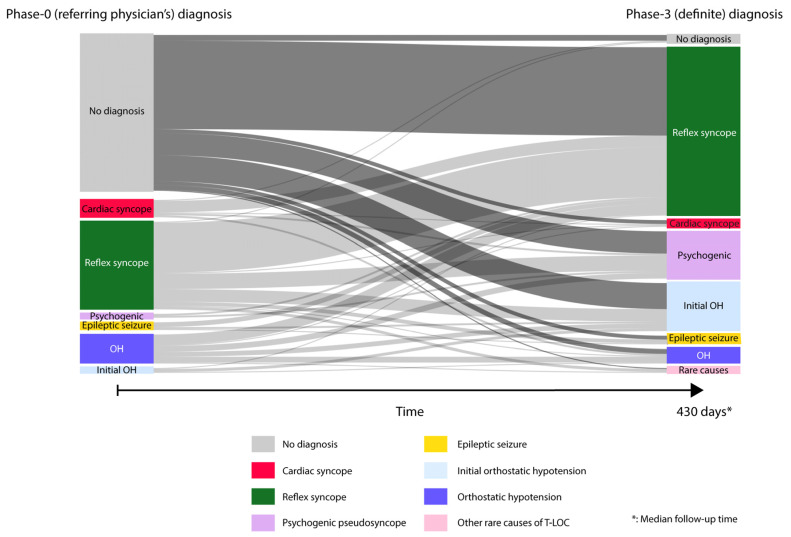
Changes in diagnoses between phase 0 (referral diagnosis) and phase 3 (reference diagnosis by critical follow-up with expert committee). Transition plot (or Sankey diagram) depicting the flow of patients with a diagnosis in phase 0 to phase 3. Coloured bars show diagnostic groups in phase 0 (left) and phase 3 (right). The size of the bars reflects the number of patients in each group. The light grey bar depicts patient flow from phase 0 to phase 3. The darker grey flow from left to right highlights the flow of patients without a phase 0 diagnosis. Flow size reflects the number of patients that correspond to the flow.

Median follow-up time was 430 days (interquartile range: 392–489 days). From the patients without a phase 0 diagnosis from the referral physician, 128 of the 134 patients (96%) received a diagnosis after phase 3. The vast majority of these patients (113 out of 134, 87%) were diagnosed in phase 3 with either reflex syncope (69%), initial orthostatic hypotension (20%), or psychogenic pseudosyncope (13%) (Figure 1). Other diagnoses were cardiac syncope (3%), classic orthostatic hypotension (4%), or epilepsy (3%) (sum > 100% due to cases with multiple causes). Importantly, only history-taking at the tertiary syncope unit (phase 1), without additional testing, yielded a diagnosis in 123 of 134 patients (93%), whereas the incremental diagnostic yield after phase 2 (autonomic function tests) was modest, with a total of 127 of 134 patients (95%) yielding a diagnosis after phase 1 or 2.

### 4.4. Diagnostic Accuracy of Different Phase 0 Diagnoses of Referring Physicians

The most frequent phase 0 diagnosis was reflex syncope (72 patients). The diagnosis of reflex syncope in phase 0 was revised in 20 patients. Additionally, 18 of the 72 patients with reflex syncope as phase 0 diagnosis received an additional diagnosis. Thus, 38 of 72 patients received an inaccurate or incomplete diagnosis. These 38 patients were mainly found to have psychogenic pseudosyncope in phase 3 (42%, 16 of 38 patients) and initial orthostatic hypotension (34%, 13 of 38 patients). 

Among the 24 patients with a classic orthostatic hypotension diagnosis at phase 0, the diagnosis could not be confirmed in 18 patients (75%) and was incomplete in one patient. These 19 patients were mainly found to have reflex syncope in phase 3 (11 of 19 patients, 58%), psychogenic pseudosyncope (six of 19 patients, 32%) and initial orthostatic hypotension (five of 19 patients, 26%). Three patients were diagnosed with psychogenic pseudosyncope in phase 0, which was inaccurate in one patient. Initial orthostatic hypotension was diagnosed at phase 0 in five patients and was inaccurate in two patients. Eight patients were diagnosed with epilepsy in phase 0, of which seven were found to be inaccurate in phase 3: five of those had reflex syncope and two initial orthostatic hypotension.

### 4.5. Diagnostic Accuracy of Cardiovascular Syncope as a Phase 0 Diagnosis

**Figure 2:**
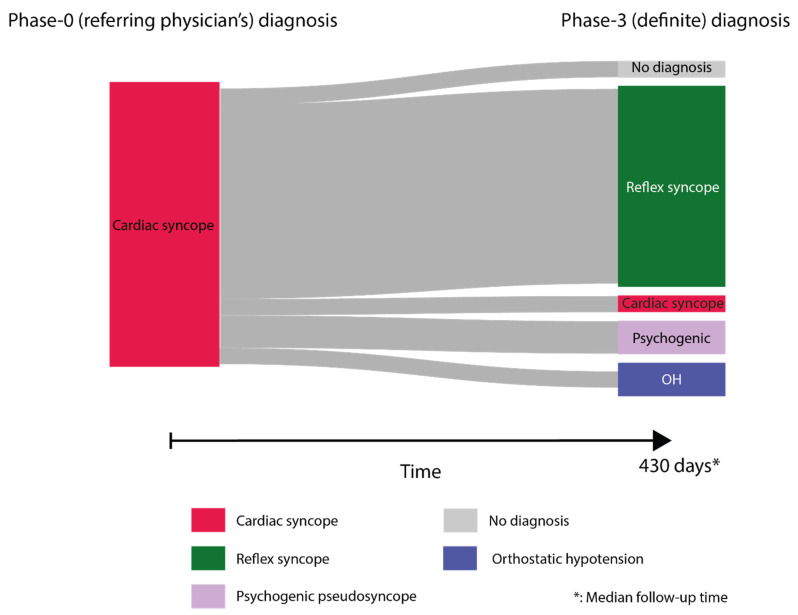
Transition plot (or Sankey diagram) depicting flow of patients with referral diagnosis of cardiac syncope (phase 0) to phase 3 diagnosis. Coloured bars indicate diagnoses. Sizes are proportional to the number of patients. Grey lines reflect patient flow from phase 0 to phase 3 diagnosis. Size reflects the number of patients in each line.

Cardiac syncope as a cause of T-LOC was the referral diagnosis (phase 0) in 18 patients, based on the presence of documented bradycardia (nine patients), syncope during walking or exercise (five patients), sudden syncope without prodromes (four patients), syncope in sitting or supine position (four patients) or palpitations preceding syncope (one patient). Remarkably, the multidisciplinary committee in phase 3 rejected cardiac syncope in 17 of 18 patients. After critical follow-up, 12 of 17 patients were diagnosed with vasovagal syncope, two with classic orthostatic hypotension and two with psychogenic pseudosyncope. In one of these 17 patients with a phase 0 diagnosis of cardiac syncope, the expert committee classified the events as ‘syncope of unknown cause’ (Figure 2). 

Three of 17 patients had a cardiac pacemaker implanted prior to the syncope unit evaluation, but syncopal episodes persisted afterwards.

## 5. Discussion

We found that nearly all patients referred to a tertiary specialized syncope unit, with or without a diagnosis upon referral, were diagnosed (phase 3) with reflex syncope, initial orthostatic hypotension or psychogenic pseudosyncope. In those that were diagnosed at referral (phase 0 diagnosis), the patients were only correctly diagnosed in 35% of the cases. These diagnoses are common, but seem to behave like “orphan” diagnoses in syncope care, resembling an important blind spot in daily clinical care. Moreover, these diagnoses underscore the importance of establishing novel approaches to non-cardiac syncope [7]. Identification or recognition of these diagnoses is important, which opens mechanism-specific therapy in order to reduce syncope recurrences in these patients. Unfortunately, these diagnoses do not seem to be part of clinical care anymore, and seem to be forgotten.

A stunning finding in our analysis was that almost every patient with a phase 0 diagnosis of cardiac syncope, of whom several patients were already implanted with pacemakers, was given an alternative diagnosis by the multidisciplinary expert panel at phase 3. Cardiac bradyarrhythmias were documented in half of those patients. Most patients with a referral diagnosis of bradyarrhythmia, during critical follow-up at our syncope unit, were found to have cardioinhibitory reflex syncope or arrhythmias that were not causally related to syncope. The most frequent phase 3 diagnosis in this group was reflex syncope and psychogenic pseudosyncope. Although this may suggest that physicians are incapable of diagnosing cardiac syncope, it should be considered that our data are a selection of patients referred for tertiary syncope care, and that conclusions on accuracy of the diagnosis cardiac syncope in primary and secondary care cannot be made from this observation. The predominance of bradyarrhythmias in the patients with inaccurately diagnosed cardiac syncope is remarkable. The ESC guidelines state that a diagnosis of arrhythmogenic syncope can be made whenever there is symptom–electrocardiographic correlation [6]. However, as also stated by the same guidelines, cardio-inhibitory reflex syncope, as an underlying cause of the bradyarrhythmia, cannot be excluded on that correlation alone. Therefore, it remains important, even in the presence of documented bradyarrhythmias, to take an extensive history and search for specific triggers of the syncopal episode. There were three patients who received cardiac pacemaker implants prior to the syncope unit evaluation, and in whom T-LOC episodes persisted. When the pacemaker is functioning correctly and T-LOC persists, a non-bradyarrhythmic cause is confirmed.

The reported prevalence of psychogenic pseudosyncope in other cohort studies ranges from 1–8% [8,9,10,11]. The relatively high prevalence of psychogenic pseudosyncope in this study may be due to underdiagnosis in secondary care. There are only a few studies in which the prevalence of initial orthostatic hypotension is separately reported [8,12,13]. The reported prevalence ranges from 3.6 to 11.2%, although recent studies showed that initial orthostatic hypotension may be much more common than previously expected [13,14]. To establish a certain diagnosis of initial orthostatic hypotension, a combination of symptoms and a significant fall in blood pressure is needed. Therefore, a continuous blood pressure monitor is needed to establish a certain diagnosis [13], as is often not available in primary and secondary care. However, in absence of a continuous blood pressure monitor, a highly likely diagnosis should be established, and subsequent treatment could be started whenever there is a typical history [13].

Thus, diagnosing psychogenic pseudosyncope and initial orthostatic hypotension primarily relies on history-taking, while ancillary tests (psychogenic pseudosyncope: home video; tilt table testing; initial orthostatic hypotension active standing test) are only needed to verify the clinical suspicion and guide therapy [14,15,16]. Of note, during the workflow in the tertiary syncope unit, a diagnosis was established in 93% of patients after history-taking only [5].

### 5.1. Education

Our results warrant additional education programs for all specialties for improvement and trust in history-taking, especially for non-cardiac diagnoses. These diagnoses are mainly the result of hampering short-term blood pressure regulation. If one does not ask the appropriate questions, clinical suspicion or awareness of these diagnoses will not be raised. The ESC guideline on syncope underscores the importance of this aspect of history-taking in the practical instructions [17]. A recent study showed that there are several barriers that impact the implementation of syncope guidelines in the emergency department [18]. The main barriers are at the healthcare professional level, reflecting the inadequate time physicians give to taking a thorough history and performing orthostatic blood pressure measurements, combined with insufficient experience/knowledge. These barriers were identified for evaluation in the emergency department. We believe, however, that time constraints also impact syncope care in outpatient clinics.

### 5.2. Lack of Structured Approach

The number of diagnostic tests performed by referring physicians prior to the consultation in our specialized syncope unit (Table 2) suggests a lack of a structured approach in most clinical practices for patients with T-LOC [4,6,19]. The diagnostic tests were generally performed by the referring physician to exclude diagnoses within their own specialty, sometimes more than once, even though the ESC guidelines specify that comprehensive diagnostic tests are only of limited value [6]. This may have resulted from the general pattern within the context of which more specialists and super-specialists are trained, and fewer generalists with a holistic view, required for tackling a common problem such as reflex syncope. A recent survey among European cardiologists found that only 65% of respondents considered orthostatic blood pressure measurement mandatory for the initial evaluation [20], while some considered other tests mandatory that are not recommended by the ESC, underlining the lack of a structured guideline-based approach among physicians. Of note, the ESC guidelines are not always driven by randomized comparisons of treatments and they rely importantly on expert opinion.

The lack of a structured approach in patients with syncope is not only supported by the low diagnostic accuracy and the multiple tests performed prior to referral, but also by the time from first T-LOC episode to a diagnosis (Table 2). Patients were diagnosed several years after their first episode. This implies a referral delay for these patients, that may have been reduced by earlier referral to a syncope unit. In terms of knowledge gaps, the knowledge gap regarding classic orthostatic hypotension is mainly attributable to misinterpretation of this term for either reflex syncope, psychogenic pseudosyncope or initial orthostatic hypotension. 

Differences of approach to syncope diagnosis exist between North America and Europe. In the former region, implantable loop recorders are preferred at an early stage. This method is useful in bradyarrhythmia, but much less in reflex syncope, as implantable loop recorders are able to only record the electrocardiogram and not blood pressure. On the other hand, at tilt testing, both ECG and blood pressure are available. However, this availability exists only in a laboratory for a short period of time. A positions sensor on the implantable loop recorders may help to distinct hypotension not related to cardio-inhibition. When a patient experiences a syncope fall, the position sensor helps confirm the vaso-depressive reflex episodes or even, more specifically, helps identify the significant cardio-inhibition occurring after a fall (late asystole), suggesting that pacing is unlikely to help [21]. Furthermore, the implantable loop recorder approach is much more expensive. A full discussion of these pros and cons has recently been published [22].

### 5.3. The Need for Nationwide Multidisciplinary Networks of Syncope Units

The question arises regarding whether the conduct of the diagnostic process can be favourably influenced, as most of the policies have been established for twenty years [23,24]. The first ESC guideline of 2001 suggested confirming a suspected diagnosis by additional diagnostic evaluation (Figure 2 in Brignole et al., 2001 [24]). Recent guidelines omit the necessity for confirmation by ancillary testing when a certain or highly likely diagnosis is established, and cardiac syncope and epileptic seizures are excluded [6]. Should efforts be made to change conduct, or should efforts be focused on changing the setting in which these patients are assessed? Blanc et al. [25] showed that education alone is not sufficient to decrease unnecessary diagnostic testing and increase diagnostic yield. Specialised syncope units in patients with unexplained syncope/T-LOC could help increase diagnostic yield and reduce tests and costs by avoiding a shot-gun approach with multiple useless tests [26]. This reinforces the importance of creating nationwide multidisciplinary networks of specialised syncope units with dedicated staff.

### 5.4. Strengths and Limitations

A strength of our study includes the use of long-term follow-up, together with expert evaluation (critical follow-up), to define the diagnostic gold standard. Another strength is the stepwise evaluation of the diagnostic performance per phase [8,19].

There are several limitations to this study. First, the referring physicians’ diagnoses were extracted from the referral letters (phase 0 diagnosis). There may have been cases where the referring physician refrained from specifying a diagnosis in the letter, even when they established one, as the patient was referred for a second opinion or diagnostic trajectory at the syncope unit anyway. Only two of 134 patients without a referral diagnosis did not report “reaching a diagnosis” as the main goal of the phase 1 consultation, which reflects that, in general, patients request a diagnosis. Surprisingly, from the 130 patients with a suggested diagnosis in the referral letter (phase 0 diagnosis), 104 (80%) considered their condition unexplained. This was distributed equally among the different phase 0 diagnoses (see Table 3). We also incorporated the patients’ perspective, indicating that they did not consider their condition explained despite the referring physician’s assigned diagnosis. This finding emphasizes the need for more time to be spent with the patient and the potential role of counselling at the initial assessment [3].

Another important limitation of this study is referral bias. In the case of a clear diagnosis, patients would not have been referred to a syncope unit. Therefore, the true diagnostic accuracy in secondary care may be much higher than found here. Although the population in this study may not reflect a representative secondary care population, the high proportion of initial orthostatic hypotension and psychogenic pseudosyncope cases suggests that those diagnoses are often missed in secondary care.

## 6. Conclusions

Patients with syncope who are undiagnosed or inaccurately diagnosed in secondary care (e.g., after visiting a cardiologist, internist or neurologist) mainly suffer from reflex syncope, initial orthostatic hypotension or psychogenic pseudosyncope. A referral diagnosis of cardiac syncope in patients referred to a tertiary syncope unit was almost never confirmed, despite the documentation of unrelated rhythm and conduction disturbances. Establishing a causal relationship between the T-LOC episodes, and cardiac rhythm and conduction disorders seems of utmost importance. A structured approach for patients suffering from syncope is yet to be widely and successfully implemented, despite clear guideline recommendations being available for more than 20 years. Access to specialized networks of syncope units should be improved to shorten the time to diagnosis and treatment.

## Figures and Tables

**Table 1:** Criteria applied for diagnosis with subjective probability level. Diagnostic criteria. This table is adapted from FAST II study (de Jong et al. [5]).

		Certain	Highly Likely	Possible
Reflex syncope	Vasovagal syncope	Syncope precipitated by pain, fear, or standing, and associated with progressive prodrome (pallor, sweating and/or nausea)	(1) Features that suggest VVS are present, but not consistent over all episodes with recognition during a positive tilt test (2) “Possible” criteria with recognition during a positive tilt test	In the presence of any features that might suggest VVS and the following observations are excluded: cardiac syncope, cOH non-syncopal LOC and subclavian steal syndrome
	Carotid sinus syndrome	Carotid sinus massage caused bradycardia (asystole) and/or hypotension that reproduced spontaneous symptoms and patients had clinical features compatible with a reflex mechanism of syncope and with specific triggers	Syncope consistent with specific triggers like head-turning, but no carotid sinus massage, is performed	Sudden syncope of unknown origin, compatible with a reflex mechanism, >40 years old, and the following observations are excluded: cardiac syncope, cOH non-syncopal LOC and subclavian steal syndrome
Orthostatic hypotension		Symptomatic abnormal BP fall and history highly suggestive of OH: syncope and presyncope are present during standing, absent while lying, and less severe or absent while sitting; a predilection for the morning; sitting or lying down must help; complaints may get worse immediately after exercise, after meals or in high temperatures; no “autonomic activation”	(1) Symptomatic abnormal BP fall and history of syncope and orthostatic complaints are possibly due to OH: not all of the features highly suggestive of OH are present (2) Asymptomatic abnormal BP fall and history of syncope and orthostatic complaints are highly suggestive of OH: syncope and presyncope are present during standing, absent while lying, and less severe or absent while sitting; a predilection for the morning; sitting or lying down must help; complaints may get worse immediately after exercise, after meals or in high temperatures; no “autonomic activation”	Orthostatic intolerance: asymptomatic fall in systolic BP and history of syncope and orthostatic complaints possibly due to OH: not all of the features of syncope always while standing, but without fall in blood pressure during orthostatic standing test, features suggesting autonomic reflex are absent
Initial OH		Frequent complaints of light-headedness, seeing black spots or (near) syncope <10 s upon active standing with disappearance of symptoms <30 s and IOH positive on active stand test	(1) Frequent complaints of light-headedness, seeing black spots or (near) syncope <10 s upon active standing with disappearance of symptoms <30 s and IOH negative on active stand test (2) “Possible” criteria and IOH on active stand test	History inconsistent, but the presence of light-headedness, seeing black spots or (near) syncope <10 s upon active standing with disappearance of symptoms <30 s, no IOH on active stand test
Cardiac syncope		Cardiac syncope was certain when a correlation between syncope and an arrhythmia (bradyarrhythmia or tachyarrhythmia) was detected in the presence of syncope	(1) All features from history are suggestive for arrhythmic syncope and (2) ECG shows high risk major features, as stated by the ESC guideline	(1) Not all features from history suggestive of arrhythmic syncope and (2) ECG shows minor criteria for high risk of cardiac syncope
Psychogenic pseudosyncope		History suggestive of PPS and recording of spontaneous attacks with a video by an eyewitness and/or witnessed an attack during AFTs	History suggestive of PPS, but no attack occurred during AFTs and no video recording of spontaneous attacks available	History inconsistent, but some features of PPS are present, no attack occurred during AFTs and no video recording of spontaneous attacks available

AFT: autonomic function test; BP: blood pressure; cOH: classic orthostatic hypotension; ECG: electrocardiogram; IOH: initial orthostatic hypotension; LOC: loss of consciousness; OH: orthostatic hypotension; PPS: psychogenic pseudosyncope; s: seconds; VVS: vasovagal syncope. Reprinted from de Jong et al. [5].

**Table 2:** Patient characteristics for those with phase 0 diagnosis and without phase 0 diagnosis. Phase 0 diagnosis is the referral diagnosis.

	Without Phase 0 Diagnosis	With Phase 0 Diagnosis	*p*-Value
*n*	134 (51)	130 (49)	
Age, median (IQR)	52 (34–66)	49 (34–63)	0.33
Total TLOC episodes, median (IQR)	6 (3–20)	5 (3–16)	0.87
Consulted specialists prior to referral median (IQR)	7 (4–10)	6 (4–12)	0.54
Last year TLOC episodes, median (IQR)	3 (1–6)	3 (1–6)	0.98
Male, *n* (%)	68 (51)	54 (42)	0.17
Diagnostic tests prior to referral, median (IQR)	10 (6–13)	9 (6–13)	0.93
Electrocardiogram, *n* (%)	120 (90)	121 (93)	0.79
Holter monitor, *n* (%)	106 (79)	101 (78)	0.67
Echocardiogram, *n* (%)	106 (79)	100 (77)	0.68
Exercise electrocardiogram, *n* (%)	99 (74)	99 (76)	0.66
Electroencephalogram, *n* (%)	79 (59)	81 (62)	0.50
X-ray thorax, *n* (%)	67 (50)	62 (48)	0.62
Computed tomography of the brain, *n* (%)	45 (34)	52 (40)	0.29
Blood pressure measurement for 24 h, *n* (%)	49 (37)	47 (36)	0.74
Cardiac magnetic resonance imaging, *n* (%)	19 (14)	21 (16)	0.66
Implantable loop recorder, *n* (%)	14 (10)	21 (16)	0.17
Carotid duplex ultrasound, *n* (%)	15 (11)	20 (15)	0.32
Head-up tilt test, *n* (%)	10 (7)	12 (9)	0.61
Myocardial perfusion scan, *n* (%)	4 (3)	4 (3)	0.97
Autonomic function tests during evaluation			
Head-up tilt test, *n* (%)	86 (64)	70 (54)	0.11
Carotid sinus massage, *n* (%)	49 (37)	35 (27)	0.12
Valsalva manoeuvre, *n* (%)	4 (3)	6 (5)	0.54
Forced-in and expiration, *n* (%)	4 (3)	6 (5)	0.54
Physical counter manoeuvres, *n* (%)	31 (23)	30 (23)	1.00
Days from first TLOC to diagnosis, median (IQR)	1167 (421–3799)	1500 (221–5291) *	0.80

* Includes only patients with an incorrect phase 0 diagnosis (see methods). h: hour; TLOC: transient loss of consciousness; IQR: interquartile range.

**Table 3:** Patients unaware of diagnosis of referring physician upon the consultation in the syncope unit.

	Phase 0 Diagnosis	Patient Unaware of Diagnosis	%
*n*	130	104	80
Reflex syncope	72	56	78
Psychogenic pseudosyncope	3	3	100
Orthostatic hypotension	29	24	83
Cardiac syncope	18	15	83
Epilepsy	8	6	75
Other	0	0	NA

NA: not applicable.

## Data Availability

The data presented in this study are openly available in FigShare at doi: 10.21942/uva.22347781.
